# What is the impact of a national postgraduate medical specialist education reform on the daily clinical training 3.5 years after implementation? A questionnaire survey

**DOI:** 10.1186/1472-6920-10-46

**Published:** 2010-06-18

**Authors:** Lene Mortensen, Bente Malling, Charlotte Ringsted, Sune Rubak

**Affiliations:** 1 Regional Hospital Viborg, Heiberg Alle 4, DK-8800 Viborg, Denmark; 2 Aarhus University Hospital Skejby, Department of Human Resources, Brendstrupgaardsvej, DK-8200 Aarhus N, Denmark; 3 University of Copenhagen and Capital Region, Centre of Clinical Education, Rigshospitalet afsnit 5404, Teilumbygningen, Blegdamsvej 9, DK-2100 Copenhagen Ø, Denmark; 4 Aarhus University Hospital Skejby, Department of. Paediatrics, Brendstrupgaardsvej, DK-8200 Aarhus N, Denmark

## Abstract

**Background:**

Many countries have recently reformed their postgraduate medical education (PGME). New pedagogic initiatives and blueprints have been introduced to improve quality and effectiveness of the education. Yet it is unknown whether these changes improved the daily clinical training. The purpose was to examine the impact of a national PGME reform on the daily clinical training practice.

**Methods:**

The Danish reform included change of content and format of specialist education in line with outcome-based education using the CanMEDS framework. We performed a questionnaire survey among all hospital doctors in the North Denmark Region. The questionnaire included items on educational appraisal meetings, individual learning plans, incorporating training issues into work routines, supervision and feedback, and interpersonal acquaintance. Data were collected before start and 31/2 years later. Mean score values were compared, and response variables were analysed by multiple regression to explore the relation between the ratings and seniority, type of hospital, type of specialty, and effect of attendance to courses in learning and teaching among respondents.

**Results:**

Response rates were 2105/2817 (75%) and 1888/3284 (58%), respectively. We found limited impact on clinical training practice and learning environment. Variances in ratings were hardly affected by type of hospital, whereas belonging to the laboratory specialities compared to other specialties was related to higher ratings concerning all aspects.

**Conclusions:**

The impact on daily clinical training practice of a national PGME reform was limited after 31/2 years. Future initiatives must focus on changing the pedagogical competences of the doctors participating in daily clinical training and on implementation strategies for changing educational culture.

## Background

Reforms in postgraduate medical education (PGME) often include major changes in the overall organisation of the education and prescriptions regarding principles of content and format of the training programmes. Recent examples of reforms are seen in many countries with the introduction of outcome-based education according to frameworks such as the roles of CanMEDS and the general competencies of the Accreditation Council for Graduate Medical Education (ACGME) [1,2]. The overall aim of these reforms is to ensure education of future physicians that are prepared to meet societal needs as well as to optimize the effectiveness of the training of future specialist doctors. Whether the reforms will meet the overall aims and goals has yet to be verified. Current evaluation and accreditation of PGME have mainly focused on adherence to the new standards for the curriculum. Whether reforms have had any impact on daily clinical training practice and the learning environment in the work-based postgraduate educational context is yet to be investigated.

The challenge of work-based education is the high demands for quality and effectiveness of the service. Thus, in organising daily clinical work routines there is a need for striking the balance of meeting the service demands and ensuring that trainees gain access to training situations that meet their learning needs. Moreover, there must be a learning environment

with a variety of clinical situations suitable for learning [3]. Supervision and feedback in daily clinical practice are of paramount importance in the work-based context of PGME [3-5]. However, the move towards including several training sites in each programme and reducing working hours of junior doctors may negatively affect the relationship and acquaintance of the junior doctors and the senior staff and hence lead to a greater need for focus on a supportive learning environment [6,7]. Proper training of the clinical trainers is thus increasingly important to ensure training during daily practice [4]. In addition, regular appraisal meetings between trainees and educational supervisor, preparation of individual learning plans and in-training assessment are strategies suggested to ensure efficient learning progress of the young doctors in the complex work-based training setting [3]. To our knowledge, no large-scale data have yet been presented on the effects of large reforms of PGME on the educational culture in clinical departments.

The purpose of this study was to examine if a major national reform of PGME has had any impact on the daily clinical training practice and work-based learning environment. As the learning experience might vary substantially across training sites [8,9] we also aimed at exploring the association between training practice and learning environment and type of hospital and speciality. The introduction of several mandatory courses was part of the initiative; we wanted to investigate if there was any effect of attending a teaching course on clinical teaching and learning strategies among respondents.

The research questions were:

1. What is the impact of a national reform of PGME on the daily clinical training practice and learning environment?

2. Is impact on training practice and learning environment related to type of training site, i.e. specialised university hospitals vs. non-university regional or general hospitals?

3. Is impact on training practice and learning environment related to type of speciality?

4. Is impact on the individual doctor's training practice related to attending courses on learning and teaching?

## Methods

The study design was a cross-sectional study of the perception among hospital doctors of issues of clinical training practice and learning environment at the start of a national reform of PGME and at 31/2 year's follow-up.

### Context of the study

The reform of PGME was a national initiative instigated by the Danish National Board of Health (DNBH). The reform included requirements of a change of content and format of PGME in line with outcome-based education using the CanMEDS' framework of seven roles and competencies: medical expert, communicator, health advocate, collaborator, manager, scholar, and professional. The specialty societies were required to outline goals and objectives accordingly and provide suggestions for clinical teaching and assessment strategies. In order to support the development of competence in the roles as communicator, scholar, and manager, the DNBH introduced mandatory courses in communication skills, learning and teaching as well as management. However, the reform did not include any requirements regarding preparation of teachers. Yet, courses on learning and teaching have long been offered to senior clinicians on a regular basis in the North Denmark Region. The content of these courses was quite similar to the new mandatory courses for junior doctors [10]. No changes in work hour or work conditions were introduced by the reform, and there were no plans for changing the hours reserved for education in the clinical setting. The PGME was decentralised to include regional hospitals as well as university hospitals in the rotations.

### Data collection

The study was a questionnaire survey among all doctors employed at hospitals in the North Denmark Region, covering about one third of the country and including approximately 3000 doctors. This region has 19 hospitals, including two specialized university hospitals, six large regional hospitals, and eleven general hospitals. All doctors were trained according to the new blueprints.

The questionnaire included items on educational appraisal meetings, preparation of individual learning plans, inclusion of training issues in the organisation of work routines, supervision and feedback, and interpersonal acquaintance among doctors. The specific questions are shown in the tables. Items were rated on a 9-point Lickert scale where one was "not at all" and nine was "very much". In addition, respondents were asked whether they had attended a course on learning and teaching. Finally, respondents were requested to indicate seniority (position as consultant, senior or junior trainee), type of hospital and type of speciality in their present employment. The questionnaire items regarding clinical training and environment were constructed by the authors on bases of a literature searching, including the reform items. In addition, items related to the structural issues of the reform were included. The questionnaire was pilot tested for interpretation of item construct and importance of content among a group of junior and senior doctors attending a standard course on learning and teaching.

An introductory letter was enclosed with the questionnaire explaining the purpose of the survey and that participation was voluntary and anonymous. In 2003, data were collected electronically from a paper edition of the questionnaire, by the software program Teleform^© ^(Teleform version 8.0, San Marcos, California, USA: Cardiff Software Inc.; 2003). A reminder was sent to non-responders after three weeks using identifiable numbers, destroyed afterwards. At follow-up in 2007, the questionnaire was sent electronically by e-mail (Enalyzer^©^). Reminders were sent electronically twice with 2-week intervals. Response time for both questionnaires was two weeks. Ethical approval was not required for this study.

### Data analysis

The data analysis excluded data set with incomplete responses regarding seniority, hospital and specialty. Types of specialty were clustered into three groups: 1) Cognitive task oriented specialities including the internal medicine specialties, paediatrics, dermatology, neurology and psychiatry, 2) Technical task oriented specialities including the surgical specialties, orthopaedics, gynaecology, otology, ophthalmology and anaesthesiology, and 3) Laboratory task oriented specialities, which included radiology, clinical biochemistry, microbiology, pathology, clinical physiology, genetics and clinical immunology.

A Mann-Whitney-test was used to compare continuous variables at reform start with 31/2-year follow-up. Response variables from both data sets were used to explore the relation between ratings of training practice and learning environment and seniority, type of hospital, type of specialty, and attendance to a course on learning and teaching among respondents. For this analysis we used ordinal categorical regression models if the assumption of parallel lines was met, i.e. the explanatory variables were equivalent across the levels of the dependent variable. Otherwise, data were analysed using multinomial logistic regression models. Background variables were year, seniority, type of hospital, type of speciality, and course attendance. The significance level was p = 0.05.

## Results

### Participants

In 2003, the questionnaires were sent to 2817 doctors of whom 2105 (75%) responded. At follow-up in 2007, we e-mailed the questionnaire to 3284 doctors of whom 1888 (58%) responded. The numbers of fully completed responses used for further analysis were 2095 at beginning of the reform and 1788 at 31/2-years follow-up. Table 1 shows the distribution of completed responses regarding seniority, type of hospital and type of speciality in 2003 and 2007, respectively.

**Table 1:** **Description of respondents **in the questionnaire sent to all hospital doctors in the North Denmark Region before (year 2003) and 31/2 years after (year 2007) instigation of a national reform on postgraduate medical education.

	Before reform	31/2 years after
**Questionnaire data**		

Number of questionnaires sent out	2817	3284

Number of responses returned	2105	1888

Number of complete responses for data analysis	2095	1788

**Characteristics of participants: N (%)**		

*Seniority:*		
Consultants	989 (47%)	1076 (57%)
Senior trainees	716 (34%)	453 (24%)
Junior trainees	400 (19%)	359 (19%)

*Hospital:*		
University hospitals	1010 (48%)	1246 (66%)
Non-university hospital	1095 (52%)	642 (34%)

*Specialty:*		
Cognitive	834 (39%)	831 (44%)
Technical	943 (46%)	812 (43%)
Laboratory	328 (15%)	245 (13%)

**Attendance at courses on learning and teaching N (%)**		

All respondents	518 (25%)	457 (25%)

Junior trainee respondents	29 (7%)	93 (26%)

Senior trainee respondents	81(19%)	108 (25%)

Consultant/specialists respondents	408 (34%)	256 (24%)

### Clinical training and educational environment

The results demonstrated that the number of respondents attending a course on learning and teaching increased (Table 1), but only among junior doctors.

Results from the survey regarding clinical training practise and learning environment in 2003 and 2007 are summarised in Table 2. Overall, the changes after the reform were very small. The value of educational appraisal meetings was rated high in both 2003 and 2007. Following the reform there was a slight but significant increase in preparations of individual learning plans. In addition, we found a small positive effect on the item 'To what extent are clinical situations used for learning?' The item 'How well do you know each other' was rated significantly lower compared to 2003. The trainees' ratings of 'receiving feedback' and 'being supervised' were rather low in 2003, 4.9 and 3.4, respectively, and did not increase following the introduction of the reform. The extent to which doctors supervise each other or give feedback on work performance did not change from 2003 to 2007.

**Table 2:** Items related to the daily clinical training practice and work-based learning environment among all hospital doctors in the North Denmark Region before and 31/2 years after instigation of a national reform on postgraduate medical education. Results are presented as mean (SD) item scores on a scale 1-9, where 9 is highest.

	Before reform	31/2 years after
Number of completed responses	N = 2095	N = 1788

What is the value of the educational appraisal meetings?	7.8(1.6)	7.7(1.9) NS
To what extent are personal learning plans for trainees prepared?	6.4(2.3)	6.8(2.2) ‡
To what extent are trainees' educational needs attended to in the organisation of daily clinical work routines?	5.1(2.3)	5.1(2.3) NS
To what extent are clinical situations used for learning?	5.0(1.9)	5.2(1.9) ‡
How well do you know each other in the department?	6.6(2.0)	6.3(2.0) ‡
How often do you give feedback to others regarding their work?	6.6(1.6)	6.6(1.7) NS
^**§ **^How often are you as a trainee receiving feedback on your work?	4.9(1.9)	4.8(2.0) NS
How often do you supervise others on their work?	5.9(2.4)	5.8(2.3) NS
^**§ **^How often are you as trainee being supervised in your work?	3.4(2.3)	3.6(2.4) NS

Significance level: ‡ p < 0.001; § = Data from trainees only; NS=No significant difference

Table 3 summarises the relations between ratings of training practice and learning environment and sub-group characteristics within the categories seniority, type of hospital, type of specialty, and attending a course on learning and teaching. For each category, it is indicated whether a sub-group characteristic significantly predicts higher or lower ratings of the questionnaire items compared to the other sub-group characteristics. In general, being a junior trainee was related to lower ratings on all aspects of training practice and learning environment compared to being a consultant or senior trainee. However, ratings of receiving feedback and being supervised were not related to being junior or senior trainee.

**Table 3:** Relation between ratings of clinical training practice and learning environment and categories of the sub-group characteristics regarding seniority, type of hospital, type of specialty, and attending a course on learning and teaching. In each category it is indicated whether a sub-group characteristic was significantly related to higher or lower ratings of the questionnaire items.

	Seniority	Hospital	Speciality	Course attendance
	**a. Consultant** **b. Senior trainee** **c. Junior trainee**	**a. University** **b. Regional** **c. General**	**a. Cognitive** **b. Technical** **c. Laboratory**	**a. Yes** **b. No**

What is the value of the educational appraisal meetings?	c < a,b ‡,†	NS	a,b < c ‡	NS
To what extent are personal learning plans for trainees prepared?	c < a,b ‡,†	NS	a,b < c †	b < a ‡
To what extent are trainees' educational needs attended to in the organisation of daily clinical work routines?	c < a ‡	a < b †	a,b < c ‡	b < a *
To what extent are clinical situations used for learning?	c < a ‡	NS	a,b < c ‡	b < a ‡
How well do you know each other in the department?	c < a ‡	a < b †	a,b < c ‡	b < a †
How often do you give feedback to others regarding their work?	c < a,b ‡,†	NS	a < c †	b < a ‡
^**§ **^How often are you as a trainee receiving feedback on your work?	NS	NS	a,b < c ‡	NS
How often do you supervise others on their work?	c < a,b ‡,*	b < a *	a,b < c *	b < a †
^**§ **^How often are you as trainee being supervised in your work?	NS	NS	a,b < c ‡	NS

Significance level: *p < 0.5 †p < 0.01 ‡ p < 0.001 NS p > 0.05; § = Data from trainees only; NS = No significant difference

Maintaining a position on one of the non-university hospitals was related to significantly higher ratings of the items 'To what extent are trainees' educational needs attended to in the organisation of daily clinical work routines?' and 'How well do you know each other in the department?' On the other hand, working in a specialised university hospital was related to significantly higher ratings regarding 'Supervising others'. Apart from that, differences in ratings were not significantly related to type of hospital.

Interestingly, working in the laboratory specialities as opposed to cognitive and technical specialities was related to higher ratings on all aspects of clinical teaching practice and learning environment.

Having attended a course on learning and teaching was associated with significantly higher ratings on most items, except for 'What is the educational outcome of the educational appraisal meetings?', and trainees' ratings of receiving feedback and being supervised.

## Discussion

This study is one of the first major studies presenting data on the effect and impact of a national PGME reform on the daily clinical training practice and learning environment in hospital departments 31/2 years after reform start. Overall, the results indicate that the reform had a small effect on some structural educational issues, but still only limited impact on daily clinical training practice and educational culture.

The impact of the reform was primarily found on two structural items, attendance to courses of learning and teaching and preparation of individual learning plans. The introduction of mandatory courses on learning and teaching for junior doctors in training was clearly reflected in the results with a large increase in number of trainees having attended such a course. However, course attendance among senior doctors decreased and corresponding to findings in other contexts, only around one quarter of the senior doctors had attended such courses after the reform [11]. Considering that in the work-based context of PGME almost all senior doctors are involved in the training of the junior doctors, the fall in course participation among senior doctors is a problem. One might speculate that the mandatory course participation should have been extended to include the seniors as well. We have previously shown that when courses on clinical teaching involved all doctors in a clinical department, it had a positive and lasting effect on training practice and educational climate [10]. This positive effect in part related to participation of all doctors from the departments, thereby reaching the "critical mass" necessary to change the educational culture and behaviour in a department. The findings of this study thus support the recommendation for a future strategy to make it mandatory for senior doctors to participate in courses on clinical training.

Regular appraisal meetings and preparation of personal learning plans became mandatory already in 1998. However, the present reform further emphasised the use of individual learning plans. The significant increase in preparation of individual learning plans following the reform might reflect that the reform had induced an increased adherence to these structural elements of the reform. However, it might also be the result of more junior doctors having attended courses on learning and teaching thus being aware of the usefulness of such plans as an essential tool for them in taking responsibility for their own training [12].

The fact that items like 'To what extent are trainees' educational needs attended to in the organisation of daily clinical work routines?' and 'To what extent are clinical situations used for learning?' only had a mean score of five before as well as after the reform indicates that structural educational initiatives fails to be effective unless the entire work-based organisation accepts and prioritises the educational responsibility in the planning of the daily clinical work. Here focus should also be on increasing the daily interaction between seniors and junior doctors. Trainees are an important part of the clinical workforce [7] and engagement in clinical work activities is pivotal for their learning [13]; supervision and feedback from seniors are crucial elements for trainees' learning in practice [7,13-15].

We found that high ratings regarding "To what extent are trainees' educational needs attended to in the organisation of daily clinical work routines?" and "How well do you know each other in the department?" were more prevalent at regional and general hospitals than at university hospitals. This finding is probably due to the smaller size of the departments. Supervision of others was higher at university hospitals, possibly due to the complexity of patients and easier access to specialists. It was apparently not related to educational culture, as university hospitals were poorer in incorporating educational needs in organisation of work routines, and trainees' ratings of receiving feedback and supervision were not related to type of hospital.

In surveys, others have found a discrepancy between consultants' and trainees' perception of the amount and quality of supervision and feedback in clinical settings [4,8]. In part, this might be due to consultants' striving at balancing supervision and development of trainees' independence [15,16]. Yet, we did not find that higher ratings were associated with being senior rather than junior trainee. Another explanation of this discrepancy might be different expectations and perceptions of what supervision is [15,17]. Part of consultants' supervision is probably backstage clinical oversight that trainees are not aware of [16,17]. However, matching expectations will be important in creating a well-balanced educational environment.

Interestingly, the laboratory specialties had higher ratings than the other specialities. This could be explained by the often small size of these departments fostering a better learning environment, similar to our findings regarding the smaller hospitals. Another reason could be that better training and supervision in laboratory specialties derive from the presence of requirements for accreditation standards regarding quality assurance of service [18] in these specialties including proper training and assessment of staff before they are given responsibility for specific service tasks. A key component of medical education reforms is the move from process to outcome evaluation [2,19] and a wide variety of assessment strategies have been suggested. It has been demonstrated that implementation of these strategies is feasible and reliable, but also that in-training assessment takes time [20]. Yet, in order for outcome assessment to be meaningful in the work-based context of PGME there is probably a need for focusing on quality of service by using assessment as a licence for trainees to independently engage in practice of specific tasks and activities [21-23]. Unfortunately, supervisors might have less focus on aspects important to the quality of care such as trainees' development of clinical skills, effective communication, and clinical decision-making in connection with cost-effective care [14]. However, our data suggest that in the work-based context, issues of quality of care might be a driving force of optimising daily teaching practice and learning environment.

It is a strength of this study that the survey included a rather large study group representing all hospital doctors in one geographical area, approximately 3000 doctors. The response rate was high (75%) in 2003, a little lower (58%) but still acceptable in 2007. The lower response rate in 2007 might be due to difficulties of keeping the list of e-mail addresses updated, especially on trainees often changing training sites and a generally lower response rate in e-mail studies.

Although the study only included a limited number of items, we doubt that the overall result of this study is due to instrumentation bias. The items in our questionnaire corresponded well with items rated of high importance in a recently developed tool, Postgraduate Educational Environment Measure (PHEEM) [24]. This international tool was subsequently validated in a Danish study [25]. Unfortunately, this tool was not available when this study was conducted. Our questionnaire was used in another study [10], successfully detecting changes in daily clinical practice. Hence, we feel confident of the validity of our instrument.

The challenge of implementing medical education reforms in the clinical context is probably not a local phenomenon, as similar findings have been reported in other contexts [26,27]. The reform process was expected to be rather slow [27] and maybe our results would have been different if the follow-up period had been longer. In order to implement reforms in the clinical work-based context wide change management strategies are necessary [26-28]. This includes involvement of all clinicians in the reform process, from leaders to trainees, and not only designated educational supervisors [27-29]. Our data suggest that the next step in implementation of the reform content must be a higher degree of involvement of senior trainers in improving the educational culture in clinical departments. In addition, accreditation bodies of postgraduate education and hospitals should increasingly focus on issues related to quality of daily clinical training practice as well as mandating courses on learning and teaching for consultants.

Future studies might explore which factors, processes and initiatives are important for successful implementation of changes in the clinical training and educational environment as well as improvements of the effectiveness of clinical trainers. Next step could be focus group interviews with senior trainers and junior trainees regarding which areas and items should be in focus in the next phase of the specialist reform.

## Conclusions

This study indicates that the national reform in PGME mainly had an effect on structural items, whereas the reform had only limited impact on the daily clinical training and learning environment. This finding stresses the importance of identifying implementation strategies for successful changes of the educational culture. It is suggested that future initiatives should increase efforts in changing the pedagogic competences and attitudes of doctors participating in clinical training thus influencing educational culture at departmental level.

## Competing interests

The authors declare that they have no competing interests.

## Authors' contributions

All authors participated in protocol and study designing. LM, BM and SR participated in data collection. LM drafted the manuscript. All authors participated in data analysis and interpretation, and contributed to critical revision of content. All authors have accepted submission of this version of the paper.

## Pre-publication history

The pre-publication history for this paper can be accessed here:

http://www.biomedcentral.com/1472-6920/10/46/prepub
